# Using Dual Microresonant Cavity and Plasmonic Effects to Enhance the Photovoltaic Efficiency of Flexible Polymer Solar Cells

**DOI:** 10.3390/nano10050944

**Published:** 2020-05-15

**Authors:** Wenfei Shen, Guoqing Zhao, Xiaolin Zhang, Fanchen Bu, Jungheum Yun, Jianguo Tang

**Affiliations:** 1 Institute of Hybrid Materials, National Center of International Joint Research for Hybrid Materials Technology, National Base of International Science & Technology Cooperation on Hybrid Materials, College of Materials Science and Engineering, Qingdao University, 308 Ningxia Road, Qingdao 266071, China; xiaolinzhang0307@126.com (X.Z.); bufanchen95@163.com (F.B.); 2 Surface Technology Division, Korea Institute of Materials Science, Changwon, Gyeongnam 51508, Korea; zhaogq@kims.re.kr

**Keywords:** flexible polymer solar cells, oxide/metal/oxide electrode, microresonant cavity, plasmonic, light absorption

## Abstract

Fabricating polymer solar cells (PSCs) on flexible polymer substrates, instead of on hard glass, is attractive for implementing the advantage and uniqueness of the PSCs represented by mechanically rollable and light-weight natures. However, simultaneously achieving reliable robustness and high-power conversion efficiency (PCE) in such flexible PSCs is still technically challenging due to poor light harvesting of thin photoactive polymers. In this work, we report a facile, effective strategy for improving the light-harvesting performance of flexible PSCs without sacrificing rollability. Very high transparent (93.67% in 400–800 nm) and low sheet resistance (~10 Ω sq^−1^) ZnO/Ag_(O)_/ZnO electrodes were implemented as the flexible substrates. In systematically comparison with ZnO/Ag/ZnO electrodes, small amount of oxygen induced continuous metallic films with lower thickness, which resulted in higher transmittance and lower sheet resistance. To increase the light absorption of thin active layer (maintain the high rollability of active layer), a unique platform simultaneously utilizing both a transparent electrode configuration based on an ultrathin oxygen-doped Ag, Ag_(O)_, and film and plasmonic Ag@SiO_2_ nanoparticles were designed for fully leveraging the advantages of duel microresonant cavity and plasmonic effects to enhance light absorbance in photoactive polymers. A combination of the ZnO/Ag_(O)_/ZnO electrode and Ag@SiO_2_ nanoparticles significantly increased the short-current density of PSCs to 17.98 mA cm^−2^ with enhancing the photoluminescence of PTB7-Th film. The flexible PSC using the optimized configuration provided an average PCE of 8.04% for flexible PSCs, which was increased by 36.27% compared to that of the PSC merely using a conventional transparent indium tin oxide electrode.

## 1. Introduction

Great progress in polymer solar cells (PSCs) has been achieved via state-of-the art fabrication techniques utilizing novel donor and acceptor materials [1,2,3]. PSCs supported on flexible transparent polymer substrates offer the merits of light weight and flexibility over inorganic solar cells [4]. The merits enable the fabrication of PSCs using facile, cost-effective roll-to-roll processes [5,6]. However, highly efficient PSCs are still challenging to obtain on polymer substrates owing to their photocurrent conversion performance (PCE) clearly being inferior than those of their inorganic counterparts. Extensive research efforts have been devoted toward enhancing light trapping and absorption in the photoactive layers of PSCs by employing various optical manipulation techniques [7,8,9,10,11,12,13].

Implementing a highly efficient transparent conductive electrode (TCE) on polymer substrates is taken into account as a primary task to resolve the issue [5,14,15,16,17,18,19,20,21]. Typical oxide-based TCEs, represented by indium tin oxide (ITO), in an amorphous phase exhibit seriously reduced electrical conductivity and optical transparency on the polymer substrates. Although notable improvements in their photoelectrical properties require applying either an elevated crystallization temperature or an increased thickness, but such options are unsuitable because of the thermal damage of heat-sensitive polymers and mechanical brittleness of thick oxides on mechanically flexible polymers [5,22]. Extensive research efforts were directed at substituting ITO with more effective TCE alternatives that exhibit improved electrical conductivity and optical transparency, high structural durability against severe mechanical deformation, long-term chemical stability, and near room temperature fabrication [7,23,24,25,26,27,28]. Patterned or irregular nanometal meshes are widely accepted as promising flexible TCEs, for instance, Jung-Yong Lee reported a > 10% PCE by using metal nanonetworks as transparent electrode [23]. However, patterned or irregular nanometal meshes are excluded from consideration because the one-dimensional geometry of the metal meshes must be further combined with conductive polymers to achieve an uniform current extraction from the entire area of PSCs. Meanwhile, continuous thin films of coinage metals (i.e., Ag, Au, and Cu) sandwiched between oxide films in an oxide/metal/oxide (OMO) configuration have drawn attention as promising TCE candidates for PSCs with their excellent photoelectric properties, mechanical flexibilities, and anticorrosion performances [5,15,16,17,29,30,31]. Recently, highly efficient OMOs using an ultrathin Ag or Cu film were developed with a special emphasis on high optical transparency. A complete continuous film geometry of such noble metals at significantly reduced thicknesses was found to form with an enhanced wetting via a simple gas-additive-induced tailoring of the growth of noble metals on oxides. The OMOs exhibited greatly improved optical transparencies in the visible spectral range (400−800 nm) while maintaining lower sheet resistances, relative to those of conventional amorphous ITOs fabricated on polymers, for instance, ZnO/Cu/ZnO with 86% average total transmittance exhibits a sheet resistance of ~10 Ω sq^−1^ much lower than that of ITO with ~30 Ω sq^−1^ sheet resistance (along with 83% average total transmittance), as result, the PCE can be over 8%. Flexible PSCs utilizing the OMOs as their front electrodes exhibited highly improved power conversion efficiencies compared to that of PSCs utilizing ITOs, while still lower than that of the same PSCs fabricated on glasses coated with crystalline ITOs. Considering the relatively low light absorption in the thin photoactive polymer layer of PSCs as the primary reason for their inferior PCEs, a further PCE improvement is readily anticipated for the PSCs by improving the light absorption in the photoactive layer via use of light-controlling techniques other than an increase in the photoactive layer that causes severe charge carrier extinction due to their low mobility and short diffusion of charge carriers in photoactive polymers.

Light manipulation techniques are effective to enhance the light trapping and absorption in photoactive layers of PSCs. Classical light manipulation techniques including antireflection structure, substrate geometry-induced trapping, plasmonic effects, optical microcavity resonance and so on, and various technologies exhibited light trapping improvements [32,33]. As one of the most studied technologies for light manipulation, the plasmonic effects of metallic nanoparticles (MNPs) are exhibited by various mechanisms, i.e., near-field enhancements (localized surface plasmon resonance, LSPR), far-field scattering, and resonant hot electron energy transfer [34]. Researchers generally agree that the plasmonic effect induced by LSPR and light scattering of metallic nanoparticles can be applied to photovoltaic devices to enhance the PCE performances [35,36]. The metallic nanoparticles can be incorporated to various layers of devices, for instance, hole transport layer, electron transport layer, and active layer. According to the mechanism of LSPR effect, indicating that the magnified electromagnetic field intensity decays with increasing the distance from nanometallic surface, incorporating MNPs into the photoactive layer is surely beneficial for the light absorption owing to the magnified electromagnetic field. As efforts to enhance the PCE of PSCs, SiO_2_-encapulated MNPs were successfully applied while avoiding exciton quenching by naked MNPs, which directly degrade the photovoltaic performance of PSCs. Meanwhile, implementing optical microresonant cavity may further improve the light absorbance in the photoactive layer by trapping optical light in the layer [35,36,37]. The enhancement in the photovoltaic performance of PSCs, either on glass or polymer substrates, was successfully achieved by realizing the virtue of the microresonant cavity effect. Jung et al. fabricated a 3D microresonant cavity system by nanopatterning processes on PET substrate and reported an improved PCE that was increased by 26.4% owing to the enhanced light absorption and charge collection efficiency. Jen et al. enhanced light absorption and photovoltaic performance by integrating the microcavity effect to flexible PSCs using Ag nanoplates on top and back electrodes [38,39]. By carefully investigation and calculations, Kim et al. presented experimental and numerical evidences indicating that the effect of microresonant cavity phenomena is confined to an interstitial medium between two metals [35] and the intensity of the optical field inside the cavity is much larger than that of the incident field [40]. Therefore, it is reasonable to believe that there are microresonant cavity phenomena between OMO electrode and metal electrodes, which can increase the light path, thus increase the light absorption.

Comprehensively implementing the dual effects of plasmonic effect and microresonant cavity effects in flexible PSCs structures, light absorption may be greatly improved by the magnified electromagnetic field and enlarged light paths, and this will provide a way to enhance the photovoltaic performances of PSCs on flexible substrates. In this work, we integrated the dual effects of the LSPR of MNPs and microresonant cavity to flexible PSCs devices. As a result, we successfully achieved the average PCEs of 8.04%, which increased by 36.27% compared to that of a flexible PSC using a conventional ITO, with a significantly improved photocurrent density. ZnO/Ag/ZnO and ZnO/Ag_(O)_/ZnO (Ag_(O)_: oxygen-doped Ag) OMO electrodes were comparatively studied by optimizing the thickness of metallic layer on the properties of transmittance, conductivity, and photovoltaic performances. In addition, the improved photovoltaic performances were ascribed to the improved total transmittance and conductivity with the incorporation of oxygen in Ag layer. Besides, the concentration of Ag@SiO_2_-NPs incorporated in the photoactive layer was optimized by enhancing the light absorption with the incorporation of Ag@SiO_2_-NPs. Improving light absorption was confirmed by clear evidences of the enhancement in the photoluminescence intensity of pure PTB7-Th film and PTB7-Th film with nanoparticles.

## 2. Experiment Details

### 2.1. Preparation of OMO Electrodes

The OMO electrodes were fabricated on a 125-μm thick, transparent poly(ethylene terephthalate) (PET) substrates (Panac Co., Ltd., Shenzhen, China) via a sequential magnetron sputtering processes at room temperature using a magnetron multigun sputtering system (A-Tech System Co., Ltd., FlexLab system 100, Taichung City, Taiwan). The ZnO layers (40 nm) were deposited from a 4 in. ZnO target (99.999% purity, Applied Science Co., Paju, Korea) at a working pressure of 4.0 × 10^−4^ pa using a radio frequency plasma power of 200 W (0.53 W cm^−2^), and no intentional thermal annealing were implemented for the ZnO layers prior to and post the deposition. Ag and Ag_(O)_ layers were deposited on the bottom ZnO layer with different thicknesses between 6 and 15 nm using a reactive sputtering process at a direct current power of 50 W (0.13 W cm^−2^). A 4 in. Ag target (99.99% purity, Applied Science Co.) was sputtered at 4.0 × 10^−4^ Pa under a mixed Ar:O_2_ gas atmosphere at a flow rate of 45:4 sccm. The oxygen concentration incorporated in Ag was determined to be about 3 at% (O/Ag atom ratio). Then, a 40 nm thick ZnO was redeposited. As for comparison, a 120 nm thick ITO electrode on flexible PET substrates were prepared using an In_2_O_3_ target (Applied Science Co.) with 10 wt% Sn. In addition, 40 nm ZnO was deposited on ITO electrode to obtain same surface condition with OMO electrodes.

### 2.2. Preparation of Polymer Solar Cell Devices

**Figure 1:**
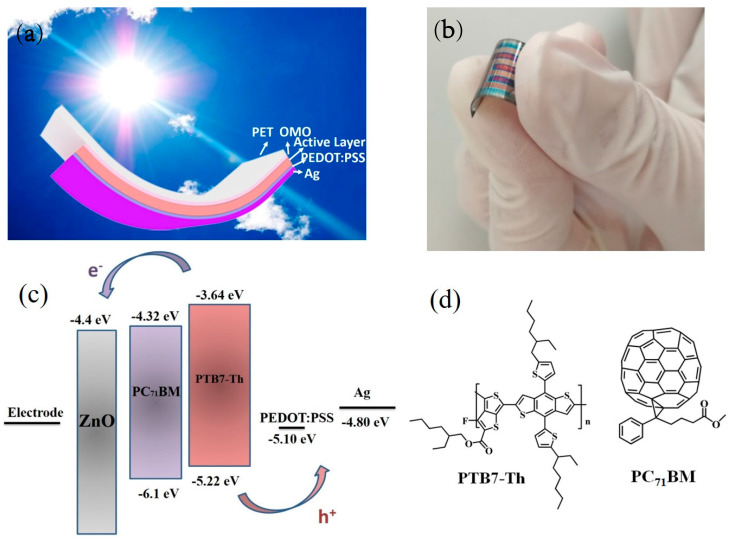
(**a**) The schematic diagram of inverted structure device, (**b**) the actual photo of flexible PSCs, (**c**) the energy levels of corresponding materials and electrodes utilized in PSCs devices, and (**d**) the chemical structure of PTB7-Th and PC_71_BM.

The inverted device structure of PET/ZnO/Ag_(O)_/ZnO/Active layer/PEDOT:PSS/Ag were shown in Figure 1a. Once the flexible, high transparent ZnO/Ag_(O)_/ZnO films were prepared on PET substrates, the photoactive polymer layer was directly coated on the OMO without adding carrier transport materials because of the electron transport performance of the ZnO top layer in the OMO electrodes. For preparing photoactive layer with metallic nanoparticle, Ag@SiO_2_-NPs were prepared by wet chemical method, with two steps. First, silver nanoparticles were synthesized at 100 °C with water as solvent, PVP as surfactant, trisodium citrate and glucose as additives, and NH_4_OH as reductant. Second, SiO_2_ shell were synthesized by modified Stöber method with ethanol and water (9:1, volume ratio) as solvent, NH_3_ as reductant, 0.011 mol TEOS diluted in 50 mL solvent, and reaction at 40 °C for 2 h. More detailed information can be found in our previous studies. Ag@SiO_2_-NPs were treated with γ-glycidoxypropyltrimethoxysilane (50 vol. % KH560) aqueous solution for 12 h to improve the dispersion of Ag@SiO_2_-NPs in the photoactive layer. The Ag@SiO_2_-NPs were washed and centrifuged 3 times, and dried in a vacuum oven at a low temperature; PTB7-Th and PC_71_BM were used as donor and acceptor materials, respectively. Ag@SiO_2_-NPs of 1.0 wt% were added in a PTB7-Th:PC_71_BM CB solution (1:1.5, 10 mg/mL for PTB7-Th, 3 vol% DIO as additive) to prepare the hybrid photoactive layer [41]. The dispersion of Ag@SiO_2_-NPs in the CB solution was improved by applying a 10-min ultrasonic treatment to the hybrid solution before its spin coating on the OMO electrode. A 3 h vacuum treatment (4.0 × 10^−2^ Pa) was implemented on the hybrid active layer to ensure the complete removal of the DIO in the photoactive layer and to fabricate the complete interpenetrating network for the photoactive layer [27]. The diluted PEDOT:PSS solution (1:10 by isopropyl alcohol) was dynamic spin coated on the photoactive layer as hole transport layer (with thickness of about 10 nm). Lastly, A 100-nm Ag electrode was thermally evaporated onto the PEDOT:PSS layer at a working pressure of 5 × 10^−4^ Pa.

### 2.3. Characterizations

The transmission electron microscopy (TEM) image of Ag@SiO_2_-NPs was obtained using a JEM-2000 Ex system. The absorption spectra of Ag@SiO_2_-NPs, PC_71_BM, and PTB7-Th were determined using a Varian Cary 50 UV−VIS spectrometer. The total transmittance spectra of PET substrate with various Ag_(O)_ thicknesses were determined using an UV−VIS spectrophotometer (equipped with integrated sphere, Cary 5000, Varian) with a normally incident radiation beam over the wavelength range of 320–800 nm. The total transmittance was equal to Transmittance/(100%−Reflection). The sheet resistances of OMO electrodes and ITO electrodes were determined using a four-point probe system (Mitsubishi Chemical Co., Tokyo, Japan, MCP-T600). The surface morphology of 10 nm Ag_(O)_ layer were observed using ultrahigh resolution field-emission scanning electron microscopy, UHR FE-SEM, (S-5500, Hitachi Co., Hong Kong, China). The current density−voltage (J−V) characteristics and the series resistance of devices were obtained by a Newport solar simulator using Keithley 2420 source measurement under AM 1.5 solar with intensity of 100 mW·cm^−2^. The external quantum efficiencies (EQE) of PSCs were determined using a certified Newport incident photon conversion efficiency (IPCE) measurement system. The photoluminescence spectra of PTB7-Th films were characterized by a FLS1000 Fluorescence Spectrometer (Edinburgh instruments Co., Livingston, UK). The active layer thicknesses can be quantified by the Veeco Dektak 150 surface profiler.

### 2.4. Simulation

The far field intensity near Ag@SiO_2_-NPs was calculated using a finite-difference time-domain (FDTD) method that was supplied by Optiwave. In the simulations, the size of core was chosen at 60 nm, while the shell thickness was chosen at 10 nm. The coated-sphere group structure was utilized; the core simulation materials was Ag (silver)—Johnson and Christy, while material used for shell was SiO_2_ (glass; with refractive index of 1.46)—Palik in the Material Database. In addition, the incident source was plane wave and corresponding wavelength was from 350 to 900 nm, and the simulation region and maximum mesh step were 100 nm × 100 nm and 1 nm, respectively.

## 3. Results and Discussion

Dual effects of microresonant cavity and plasmonics were implemented by flexible PSCs in a structural configuration of PET/OMO/active layer/PEDOT:PSS/Ag (Figure 1a). The inverted structure of PSCs was considered in this study because of their multiple advantages. It is accepted to be more stable than the normal structure of PSCs with the virtue of the Ag electrode exhibiting a high work function [42]. Furthermore, the photocurrent density of inverted PSCs can be increased by applying the metal oxide (ZnO, TiO_2_, etc.) as an electron transport layer that exhibits a transmittance much higher than that of PEDOT:PSS in normal PSCs [5,43]. Here, no additional metal oxide layer is needed on the OMO electrode for transporting electron carriers because of the existence of the top ZnO layer in the OMO electrode. The superior mechanical flexibility of the PSC based on the OMO electrode is illustrated in Figure 1b. The energy levels of materials used in the PSC are illustrated in Figure 1c, and considering the relative thickness of ZnO layer, the work function of OMO electrode is difficult to measure, only ZnO energy level is demonstrated. When the chemical structures of PTB7-Th and PC_71_BM are schematically demonstrated in Figure 1d, the lowest unoccupied molecular orbit (LUMO) energy levels of PTB7-Th, PC_71_BM, and ZnO are −3.64, −4.32, and −4.4 eV, respectively. The appropriate difference between the energy levels of those materials provides favorable circumstances promoting the dissociation of excitons and the transport of electrons [44]. The highest occupied molecular orbit (HOMO) energy levels of PC_71_BM, PTB7-Th, PEDOT:PSS, and Ag which are approximated to be −6.1, −5.22, −5.1, and −4.8 eV [45], respectively, also provide favorable circumstances promoting the dissociation of excitons and the transport of holes [44,46]. The proposed structural configuration of PSCs provides an excellent chance of highly efficient photon-to-electron conversion [47].

**Figure 2:**
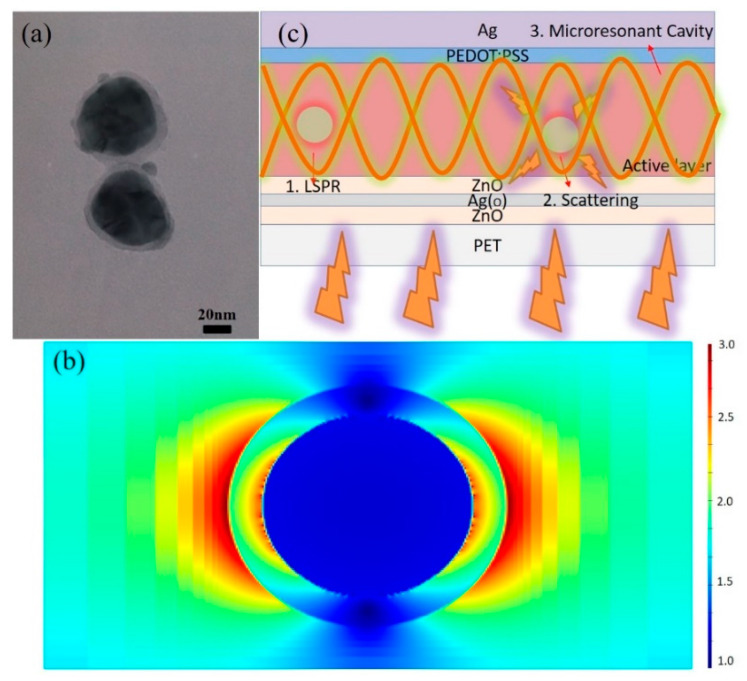
(**a**) The TEM image of Ag@SiO_2_-NPs, (**b**) the FDTD simulation result of Ag@SiO_2_-NPs, and (**c**) the enhancing mechanism by LSPR, scattering, and microresonant cavity effects.

The morphology of Ag-NPs was characterized because the plasmonic and scattering effects of metallic nanoparticles were strongly depending on the shape and size of the nanoparticles. Figure 2a shows the TEM image of Ag@SiO_2_-NPs that indicates total size of 65–80 nm with the core size of about 60–70 nm and the SiO_2_ shell thickness of about 5–10 nm. We previously demonstrated that the contact of bare metallic nanoparticles with photoactive materials could cause exciton quenching. However, the existence of dielectric material, SiO_2_, could prevent effectively such the degradation in the photovoltaic performance by blocking the “hot electrons” from the contact to the photoactive materials [48]. The LSPR effect can be presented by the magnified electromagnetic field surrounding the nanoparticles, and the magnified electromagnetic field surely decays with an increased distance to nanoparticle surface. The computational results are shown in Figure 2b. Here, the LSPR effect was directly determined by FDTD simulations. The simulated far field intensity of Ag@SiO_2_-NPs is shown in the color image in which the colors represent the magnified electromagnetic field intensity [48]. The results indicated that the intensity of electromagnetic field can be magnified up to two to three times with the existence of SiO_2_ that does not block the magnified electromagnetic field. However, the intensity decays with increasing the distance from the surface of NPs. The magnified electromagnetic field extending to surroundings promotes the light absorption of the materials surrounding the Ag@SiO_2_-NPs. Considering the existence of incorporated Ag@SiO_2_-NPs, the thin Ag_(O)_ layer, and the back Ag anode electrode in this work, it is reasonable to believe that there are three kinds of effects which will influence the light absorption or light path, as shown in Figure 2c, (1) LSPR effect, (2) scattering effects, and (3) microresonant cavity effects [33,49]. For LSPR effect, the light can be confined to a small space and the electromagnetic field can be magnified to several times, which will benefit the light absorption of donor/acceptor materials surrounding the NPs. As shown in Figure 2c, the light can be scattered by the NPs, which will increase the light path in the active layer, thus reasonably increase the light absorption. In addition, the existence of Ag_(O)_ thin layer (~7.5 nm) and the back Ag anode electrode (~100 nm) promise the presence of microresonant cavity, as shown in Figure 2c; although partial light go through thin Ag_(O)_ layer, most of light will oscillate in active layer between front and back electrodes, which greatly increase the light path, thus increase the light absorption. Considering the relatively small size of NPs, it is believed that the LSPR effect and microresonant cavity effect dominate the contributions of light absorption increments. Therefore, the light absorption of active layer in devices can be greatly increased with LSPR, scattering, and microresonant cavity effects [35,48].

**Figure 3:**
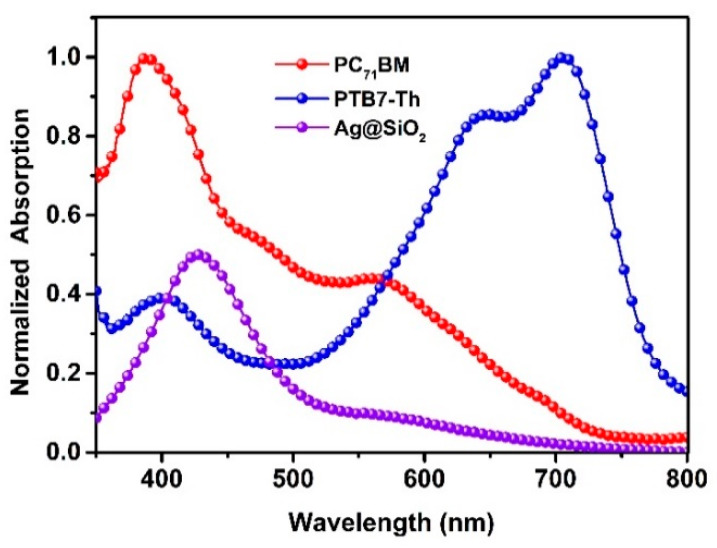
Light absorptions of Ag@SiO_2_-NPs solution in ethanol, and PTB7-Th solid film, PC_71_BM solid film.

LSPR effect occurs when the incident light frequency is equal to the electron oscillation frequency on the metallic nanoparticle surface. To characterize the resonant light wavelength, UV-VIS absorptions of Ag@SiO_2_-NPs along with donor/acceptor materials were done, as shown in Figure 3. The relative strong light absorption for PTB7-Th is in the wavelength range of 600–750 nm. The light absorption peak for PC_71_BM is 390 nm, and the light absorption decay with the increase of wavelength. The LSPR wavelength is about 440 nm, and the peak is not relatively wide, which is beneficial in the light absorption. Apart from the near-field enhancements that are caused by the magnified electromagnetic field, researchers also found that the light-induced excitons can combine with the resonance plasmons, which induces the light absorption ability go up to several times. Therefore, the plasmonic effect not only induces more photons confined in a small volume to increase the light absorption of materials surrounding metallic nanoparticles but also increases the light absorption ability of materials surrounding metallic nanoparticles. So, most importantly, the incorporation of Ag@SiO_2_-NPs will increase the light absorption ability of photoactive materials in the range of 400–500 nm, thus induce an enhancement of photovoltaic response in this range.

**Figure 4:**
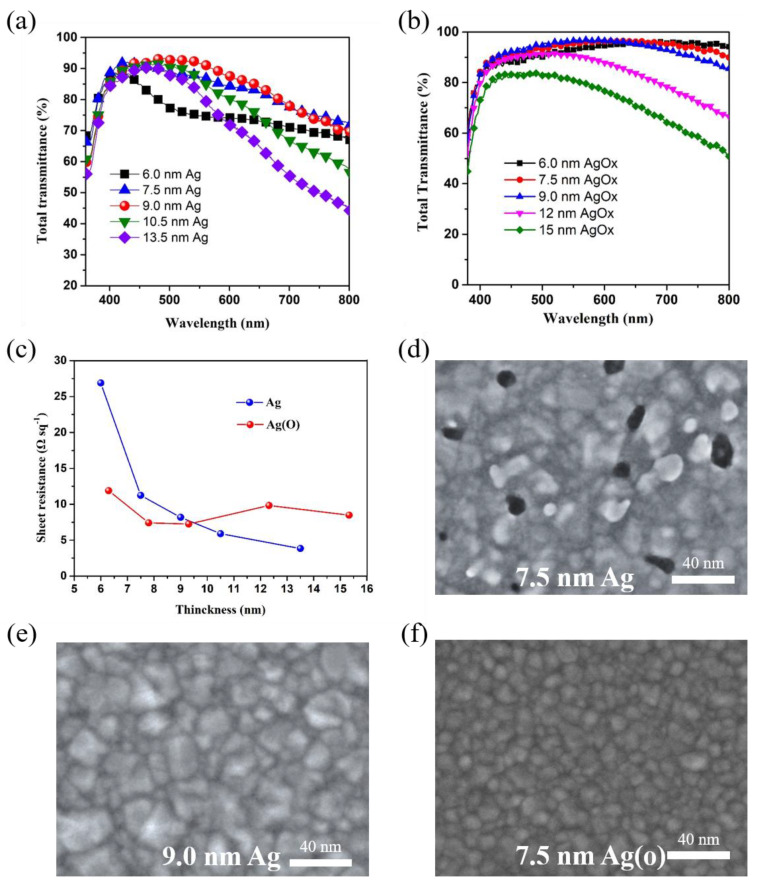
(**a**) Total transmittance of PET substrate with ZnO/Ag/ZnO OMO electrode for various Ag thicknesses, 6.0, 7.5, 9.0, 10.5, and 13.5 nm; (**b**) Total transmittance of PET substrate with ZnO/Ag_(O)_/ZnO OMO electrode for various Ag_(O)_ thicknesses, 6.0, 7.5, 9.0, 12, and 15 nm; (**c**) sheet resistance variation as a function of different metallic layer thickness; and SEM images of Ag (7.5 nm) (**d**), Ag (9.0 nm) (**e**), and Ag_(O)_ (7.5 nm) (**f**).

To get higher photocurrent density and better charge extraction efficiency for devices on flexible substrates, relatively high transmittance and low sheet resistance are required for the OMO electrodes. In addition, in order to trap more photons by the microresonant cavity effects in active layer to realize better photovoltaic performance, the light transmittance should be maximized, which greatly depends on the thickness of metallic layer in OMO electrodes [50]. Therefore, first, Ag and Ag_(O)_ thicknesses were optimized to systematically study the light transmittance property. To characterize the transmittance of OMO electrodes on PET substrates, total transmittances of 360–800 nm including scattering with various Ag and Ag_(O)_ thicknesses were done, as the results are shown in Figure 4a,b. From Figure 4a, it can be seen that the transmittance of the ZnO/9.0-nm Ag/ZnO TCE reached 91.5% at 550 nm. Ag thickness between 6 and 7.5 nm shows a lower transmittance than that for 9.0 nm, which can be ascribed to the light absorption and light scattering effects that are caused by the bigger nanoparticles of the uncontinuous thin film. The transmittance dramatically decreased from the maximum value with further increase in the thickness from 9.0 nm. The optical loss was especially significant in the longer wavelengths over 600 nm mainly due to strong increase in the reflection with increase in the thickness. In Figure 4b, the total transmittance of Ag_(O)_ OMO electrodes on PET substrates with 7.5 and 9.0 nm thicknesses is higher than that of 8 nm Ag_(O)_ OMO electrode on PET substrate in the wavelength range of 400–650 nm [51]. In the wavelength range more than 650 nm, the total transmittance of 7.5 and 9.0 nm OMO electrodes is much lower than that of 8 nm Ag_(O)_ OMO electrode on PET substrate. Besides, the total transmittance of 12 nm Ag_(O)_ OMO electrodes on PET substrates decrease greatly in the wavelength range of 550–800 nm, and the total transmittance of 15 nm Ag_(O)_ OMO electrodes on PET substrates is much lower than that of substrates with thinner Ag_(O)_ layer, which is caused by the photon reflection of thick Ag_(O)_ layer. Generally, the overall total transmittance of Ag_(O)_ OMO electrodes is better than that of Ag OMO electrodes, which can be confirmed by the average transmittance in 400–800 nm wavelength range in Table 1 and Table S1. For instance, the highest average value (89.28%) of Ag OMO electrodes with 9.0 nm Ag is much lower than that of Ag_(O)_ OMO electrode (93.67%) with 7.5 nm Ag_(O)_. Figure 4c shows that the sheet resistances varies with the Ag and Ag_(O)_ thickness; from the results, it can be seen that the sheet resistance of the ZnO/Ag/ZnO electrode decreased with the increase of Ag thickness, the slops slowed down and ZnO/9.0-nm Ag/ZnO electrode reached below 10 Ω sq^−1^. Although the sheet resistances of OMO electrodes with various Ag_(O)_ thickness change a little, the values are around 10 Ω sq^−1^ that promise a good electric property. The optimal optoelectrical properties of the Ag and Ag_(O)_ OMO TCE at metallic layer thickness of 9.0 nm surely exceed that of amorphous ITO film electrode, which are usually available by room temperature sputtering processes on heat-sensitive PET substrates, exhibiting about 85% at about 30 Ω sq^−1^. The FE-SEM images in Figure 4d–f indicated that such optoelectrical properties were attributed to the formation of a completely continuous film geometry at the thickness. It seems that the Ag thickness of 9.0 nm other than 7.5 nm is the minimum thickness for forming such a continuous film by completely filling holes, which surely improves electron transport [52]. However, the small amount of oxygen incorporation is good for the construction of continuous thin Ag_(O)_ layer on ZnO layer, and the continuous film can be formed in a lower Ag_(O)_ thickness of 7.5 nm. Even with same metallic thickness, oxygen incorporation can induce a more homogenous layer with small domain size, which causes smaller light loss and better transmittance [5].

**Figure 5:**
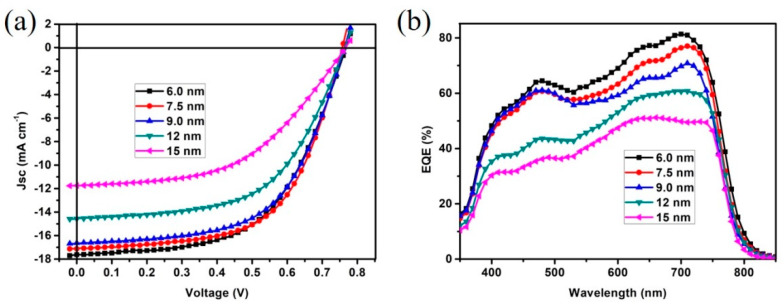
(**a**) I–V curves of devices with ZnO/Ag_(O)_/ZnO electrodes for different Ag_(O)_ thickness, (**b**) corresponding EQE spectra of devices with different Ag_(O)_ thickness.

The high transmittance and low sheet resistance promise good photovoltaic performances for flexible PSCs. To characterize the performance of OMO electrodes, flexible PSCs devices were fabricated and corresponding I-V and EQE measurements were characterized. The photovoltaic parameters of devices with 6.0, 7.5, 9.0, 12, and 15-nm thick Ag_(O)_ layers are demonstrated in Table 1, and the corresponding I-V curves of devices are shown in Figure 5a. The photovoltaic parameters of devices with 6.0, 7.5, 9.0, 10.5, and 13-nm thick Ag layers are demonstrated in Table S1, and the corresponding I-V curves of devices are shown in Figure S1. The photovoltaic performances of ZnO/Ag_(O)_/ZnO OMO electrodes are obvious better than that of ZnO/Ag/ZnO OMO electrodes, which can be ascribed to better optical and electric properties demonstrated above. Carefully checking the results, it can be seen that the optimized Ag_(O)_ thickness is 7.5 nm, with which the related devices exhibited the highest average PCE of 7.79%, with relatively high average short current density (*Jsc*) of 17.12 mA cm^−2^ and highest average fill factor (FF) of 59.96%, because of the relatively high total transmittance and low series resistance. Besides, the series resistance value reflects internal resistance of devices, the lower value indicated better junction contact and lower self-exploitation. Therefore, from Table 1, it can be seen that the values of FF are in accordance with that series resistances, which means lower series resistance promises better devices structure. The devices with 6.0 and 9.0 nm Ag_(O)_ layers showed good photovoltaic performances too. Devices with 6.0 nm Ag_(O)_ layer exhibited an average PCE of 7.65%, with highest average *Jsc* of 17.62 mA cm^−2^, because of the highest total transmittance. For devices with Ag_(O)_ layers more than 9.0 nm, i.e., 12 and 15 nm, the photovoltaic performances decrease greatly, mainly due to the greatly decreased photocurrent, which are induced by the reduced total transmittance value. The photovoltaic performances can be verified by the EQE measurement, for instance, by calculating EQE curve area, the calculated *Jsc* can be obtained [46,53,54]. Figure 5b shows the EQE curves of devices with various Ag_(O)_ thickness, and from the result, it can be seen that the device with 6.0 nm Ag_(O)_ layer shows highest EQE value, the calculated *Jsc* is 17.45 mA cm^−2^, which in accordance with that of value obtain by *I*−*V* measurement. In addition, as Figure 5b and Table 1 showed, the EQE values and calculated *J*sc decrease with the increasing Ag_(O)_ thickness, which is in accordance with the changes of total transmittance. To investigate the photovoltaic performances of Ag_(O)_-based OMO electrode, PET/ITO (120 nm)/ZnO (40 nm) electrodes were prepared and corresponding devices were fabricated, as the photovoltaic parameters illustrated in Table 1. Carefully checking the photovoltaic parameters, it is found that the greatly enhanced PCE of OMO devices are mainly caused by the enhanced *Jsc* and FF, with average *Jsc* increasing from 13.70 to 14.56 mA cm^−2^, improved by 6.27% and with average FF increasing from 55.13% to 56.70%, improved by 2.84%. Considering that both of the two electrodes contain relatively thick ZnO as top layer, the work function of Ag and ITO are well modified to form good interface contacts. Therefore, the slightly enhanced FF might be caused by the improved sheet resistance. It is more reasonable to believe that the greatly enhanced *Jsc* is caused by the microresonant cavity effects, with the evidence that lower average total transmittance and higher *Jsc* is present in OMO devices compared with that of ITO devices.

**Figure 6:**
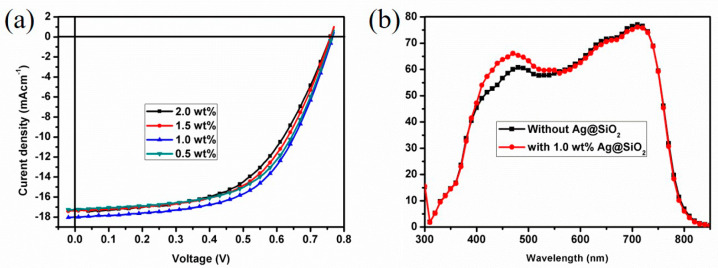
(**a**) I-V curves of devices with different weight percent Ag@SiO_2_-NPs incorporation, (**b**) EQE curves of device with optimized Ag@SiO_2_-NPs incorporation and that of the reference device.

To further increase the photovoltaic performance of ZnO/Ag_(O)_/ZnO-based flexible PSCs, Ag@SiO_2_-NPs were introduced to the PTB7-Th:PC_71_BM blend active layers to utilize the plasmonic effects of metallic nanoparticles, and the incorporation concentration of Ag@SiO_2_-NPs was optimized by constructing devices with various concentrations, as Table 2 illustrated detailed photovoltaic parameters and Figure 6a showed the corresponding I–V curves. From the results, it can be seen that the optimized Ag@SiO_2_-NPs concentration is 1.0 wt%; with the optimized concentration, the average PCE can be as high as 8.04%, with highest average *Jsc* as 17.98 mA cm^−2^ and relatively high average FF of 58.40%. Compared with the PCE of ITO devices shown in Table 1, the PCE of devices with1.0 wt% Ag@SiO_2_-NPs increased by 36.27% because of the dual effects, plasmonic effect and microresonant cavity effect. Similar enhancements can also be achieved with ZnO/Ag/ZnO OMO electrodes, as results shown in Table S2 and Figure S2. Besides, in Table 2, with higher concentrations, the *Jsc* and FF of devices are relatively lower than that of optimized devices, which may be caused by the destruction of the internetwork of active layer by the large aggregations due to the high NPs incorporation, which has been illustrated in detail in our previous papers. The destroyed nanostructure can be confirmed by the *Rs* values, as with lower concentrations, the Rs value is higher than that of devices with higher concentrations, which indicates that as the concentration increases, the internal series increases and the working efficiency decreases. In addition, with lower concentration, i.e., 0.5 wt%, *Jsc* decreases while the FF value remains high, as the average value of 58.49%. There is no difference for Voc values of devices with various Ag@SiO_2_-NPs concentrations, which indicates that the incorporation of Ag@SiO_2_-NPs causes no electric changes, because of the encapsulation of metallic nanoparticles by dielectric material (SiO_2_). Our previous research [48] have proved that without SiO_2_ encapsulation, the bared Ag nanoparticle will induce great decrease of photovoltaic performances, with greatly decreased *Voc*, *Jsc*, and *FF* values. This phenomenon is caused by excitons quenching, which is induced by the incorporated Ag nanoparticles acting as the recombination center to quench the excitons. The dielectric material SiO_2_ encapsulation can perfectly prevent the excitons quenching without causing electric changes, as utilizing their plasmonic effects during main effective charge separation working mechanism. However, it is unavoidable that the incorporation of Ag@SiO_2_-NPs slightly affects the interpenetrating nanostructure of active layer, which may cause the changes of FF and Rs. Figure 6b shows the EQE spectra of devices with or without optimized Ag@SiO_2_-NPs concentrations. As a result, with Ag@SiO_2_-NPs, the EQE spectrum of device shows enhanced values in the wavelength range of 400–500 nm, which is in accordance with the LSPR wavelength range. Therefore, it is reasonable to believe that the enhancements are caused by the LSPR effect of nanoparticle. In addition, considering the relatively large Ag@SiO_2_-NPs size, there are the light scattering effect of NPs, which is good for the enlarging of the light paths, thus good for the light absorption. 

**Figure 7:**
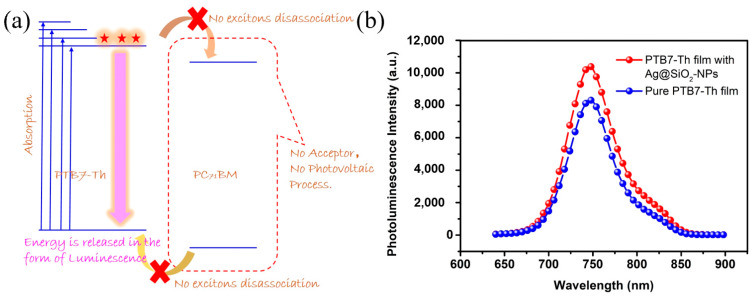
(**a**) Schematic diagram of the luminescence process without the existence of PC_71_BM and (**b**) photoluminescence spectra of pure PTB7-Th film and PTB7-Th film with Ag@SiO_2_-NPs; excitation wavelength was 612 nm.

It is difficult to demonstrate the incorporation of Ag@SiO_2_-NPs caused the enhanced light absorption directly by one characterization. However, it can be proved by the photoluminescence (PL) measurement of PTB7-Th film with 1 wt% Ag@SiO_2_-NPs and pure PTB7-Th film [40,54]. The reasons that PL measurement verifies the light enhancement can be illustrated by Figure 7a. As seen, PTB7-Th absorbs photons-induced electrons excited to the excited states, and go to the relatively stable the first excited state (LUMO). Most of electrons on first excited state of donor material (PTB7-Th) will transfer to the first excited state of acceptor material (PC_71_BM) if existed, and there is photovoltaic process. Without acceptor materials, electrons on first excited state of donor material (PTB7-Th) will go back to the ground state by the energy release of luminescence or heat. Therefore, it is reasonable to believe that the PL intensity is proportional to the absorption of light and the PL measurement can be used to verify the enhanced light. To guarantee the reliability of the PL results, the concentration of donor polymer, solvent, and spin coating speed were fixed; as a result, the thickness of pure PTB7-Th film and PTB7-Th film with 1 wt% Ag@SiO_2_-NPs were characterized to be 102 and 99 nm. Figure 7b shows the PL intensity results of pure PTB7-Th film and PTB7-Th film with 1 wt% Ag@SiO_2_-NPs; the PL intensity of PTB7-Th film with 1 wt% Ag@SiO_2_-NPs is much higher than that of pure PTB7-Th film. Considering the similar film thickness, the enhanced PL intensity is caused by enhanced light absorption. Therefore, it can be verified that the incorporation of Ag@SiO_2_-NPs indeed cause the enhanced light absorption of active layers, by the LSPR effect and light-scattering effect.

**Figure 8:**
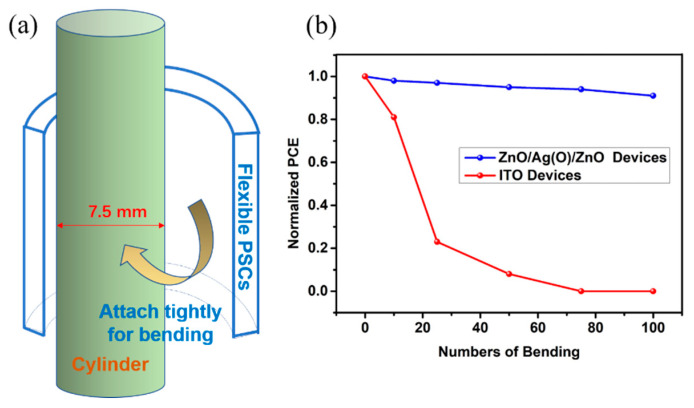
(**a**) Schematic diagram of bending test and (**b**) changes in the normalized PCE of PSC devices utilizing ZnO/Ag_(O)_/ZnO and ITO electrodes as a function of number of bending.

Rollability is an important property for flexible PSCs along with the photovoltaic performances. In order to evaluate the rollability of flexible PSCs with ZnO/Ag_(O)_/ZnO OMO electrodes, the bending tests were implemented by bending the PSC devices on a cylinder with a diameter of 7.5 mm, as schematically illustrated in Figure 8a [52]. The PCE value was recorded after 10, 25, 50, 75, and 100 times bending, as shown in Figure 8b. The flexible PSC using a ZnO/Ag_(O)_/ZnO electrode exhibited excellent rollability by maintaining over 90% performance after 100 times bending. However, the same device using an ITO electrode exhibited much poorer rollability. After 10 times bending, the normalized PCE decreased greatly.

## 4. Conclusions

High transmittance and low sheet resistance OMO electrodes were implemented as the electrode materials on flexible PET substrates. By small amount of oxygen, continuous metallic film can be formed with lower thickness in Ag_(O)_ OMO electrodes other than Ag OMO electrodes, which result in much higher average total transmittance and lower sheet resistance. As a result, with 7.5 nm Ag_(O)_, the devices can obtain high *Jsc* and high FF, thus high PCE. With OMO electrode and back metal electrode, microresonance cavity effect plays an important part in trapping light between OMO and back electrode, which greatly increases the light path. To further enhance the photovoltaic performance of flexible PSCs, Ag@SiO_2_-NPs were incorporated to active layer to utilize the plasmonic effect. With optimized concentration of Ag@SiO_2_-NPs, the average PCE can be enhanced by 36.27%–8.04%, with enhanced Jsc value of 17.98 mA cm^−2^. The enhanced EQE wavelength range is in accordance with that of LSPR wavelength range indicating that enhancement is induced by the LSPR effect. The PL measurement verified that the incorporation of Ag@SiO_2_-NPs indeed causes the enhanced light absorption. Besides, the flexible devices with OMO electrodes exhibit much better rollability, compared with that of ITO devices. All in all, the incorporation of Ag@SiO_2_-NPs to OMO-based flexible PSCs achieved good photovoltaic performances, induced by the plasmonic and microresonance cavity effects, which can be utilized in the future commercialization of flexible PSCs.

## Figures and Tables

**Table 1:** Ag_(O)_ thickness optimizing of PET/ZnO/Ag_(O)_ (6.0, 7.5, 9.0, 12, and 15 nm)/ZnO substrates.

TE Type	Ag(O) Thickness (nm)	Average T (%) in 400–800 nm	Voc (V)	*Jsc* (mA cm^−2^)	FF (%)	PCE (%)	Calculated *Jsc* from EQE (mA cm^−2^)	Rs (Ω cm^2^)
ZnO/Ag_(O)_/ZnO	6.0	92.90	0.77 ± 0.01	17.62 ± 0.25	56.42 ± 0.56	7.65 ± 0.11	17.45	6.57
ZnO/Ag_(O)_/ZnO	7.5	93.67	0.76 ± 0.01	17.12 ± 0.32	59.96 ± 0.42	7.79 ± 0.13	17.04	3.07
ZnO/Ag_(O)_/ZnO	9.0	92.96	0.76 ± 0.01	16.64 ± 0.37	58.49 ± 0.63	7.40 ± 0.16	16.26	3.58
ZnO/Ag_(O)_/ZnO	12	83.60	0.76 ± 0.01	14.56 ± 0.42	56.70 ± 0.78	6.27 ± 0.21	13.59	5.79
ZnO/Ag_(O)_/ZnO	15	72.79	0.77 ± 0.01	12.07 ± 0.39	50.34 ± 0.69	4.65 ± 0.19	11.51	10.11
ITO/ZnO	0	85.66	0.77 ± 0.01	13.70 ± 0.22	55.13 ± 0.53	5.90 ± 0.24		9.24

The average photovoltaic values were obtained from 10 devices.

**Table 2:** Ag@SiO_2_ concentration optimizing in active layer on PET/ZnO/Ag_(O)_ (7.5 nm)/ZnO substrates.

Ag @SiO_2_ conc.	Voc (V)	*Jsc* (mA cm^−2^)	FF (%)	PCE (%)	Rs (Ω cm^2^)
2.0 wt%	0.76 ± 0.01	17.25 ± 0.24	55.41 ± 0.71	7.26 ± 0.19	9.67
1.5 wt%	0.76 ± 0.01	17.39 ± 0.36	56.88 ± 0.84	7.51 ± 0.31	6.34
1.0 wt%	0.77 ± 0.01	17.98 ± 0.28	58.40 ± 0.52	8.04 ± 0.21	3.27
0.5 wt%	0.76 ± 0.01	17.22 ± 0.29	58.49 ± 0.44	7.69 ± 0.16	4.06

The average photovoltaic values were obtained from 10 devices.

## Supplementary Materials

The following are available online at https://www.mdpi.com/2079-4991/10/5/944/s1, Figure S1: (a) I-V curves and corresponding EQE spectra of PSCs using ZnO/Ag/ZnO electrodes with different Ag thicknesses, Figure S2. (a) I-V curves of PSC devices using different concentrations of Ag-SiO_2_-NPs incorporated in the photoactive layer, (b) Comparison of EQE spectra of a PSC device applying the optimized incorporation of NPs and a NP-free PSC device, Table S1: Photovoltaic performances of PSCs fabricated using PET/ZnO/Ag/ZnO substrates with different Ag thicknesses, Table S2: Changes in photovoltaic performances of flexible PSCs using a ZnO/7.5-nm/ZnO TCE as a function of the concentration of Ag-SiO_2_-NPs.
