# Supplementary material for: Using Dual Microresonant Cavity and Plasmonic Effects to Enhance the Photovoltaic Efficiency of Flexible Polymer Solar Cells
Source: Nanomaterials (Basel). 2020 May 15;10(5):944. doi: 10.3390/nano10050944 (PMC7279274; doi:10.3390/nano10050944)
Supplement: Supplementary file 1 [file nanomaterials-10-00944-s001.pdf]

Table S1. Photovoltaic performances of PSCs fabricated using PET/ZnO/Ag/ZnO substrates with different Ag thicknesses.

Ag thickness (nm)	Average T (%) in 400–800 nm	Voc (V)	Jsc (mA cm^{-2})	FF (%)	PCE (%)	Calculated Jsc from EQE (mA cm^{-2})
6	80.25%	0.72 ± 0.01	13.81 ± 0.21	56.71 ± 0.44	5.64 ± 0.13	13.52
7.5	87.64%	0.74 ± 0.01	16.10 ± 0.34	60.92 ± 0.52	7.26 ± 0.21	15.43
9	89.28%	0.75 ± 0.01	15.23 ± 0.27	59.68 ± 0.49	6.82 ± 0.17	14.78
10.5	81.06%	0.72 ± 0.01	14.03 ± 0.39	58.23 ± 0.32	5.88 ± 0.20	13.41
13.5	76.43%	0.73 ± 0.01	11.17 ± 0.18	58.84 ± 0.47	4.80 ± 0.12	10.51

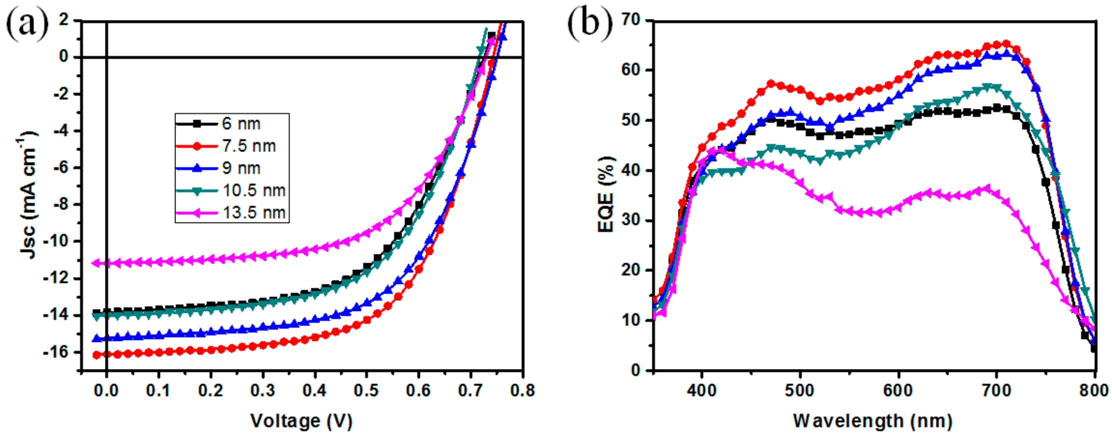

Figure S1. (**a**) I-V curves and corresponding EQE spectra of PSCs using ZnO/Ag/ZnO electrodes with different Ag thicknesses; (**b**) corresponding EQE spectra of devices with different Ag thickness.

Table S2. Changes in photovoltaic performances of flexible PSCs using a ZnO/7.5-nm/ZnO TCE as a function of the concentration of Ag–SiO_2 NPs.

Ag -SiO_2 concentrations	Voc (V)	Jsc (mA cm^{-2})	FF (%)	PCE (%)
2.0 wt%	0.74 ± 0.01	16.83 ± 0.34	56.61 ± 0.52	7.11 ± 0.15
1.5 wt%	0.75 ± 0.01	17.24 ± 0.31	57.74 ± 0.65	7.46 ± 0.23
1.0 wt%	0.75 ± 0.01	17.57 ± 0.22	57.92 ± 0.57	7.60 ± 0.21
0.5 wt%	0.74 ± 0.01	16.71 ± 0.38	58.65 ± 0.49	7.29 ± 0.19

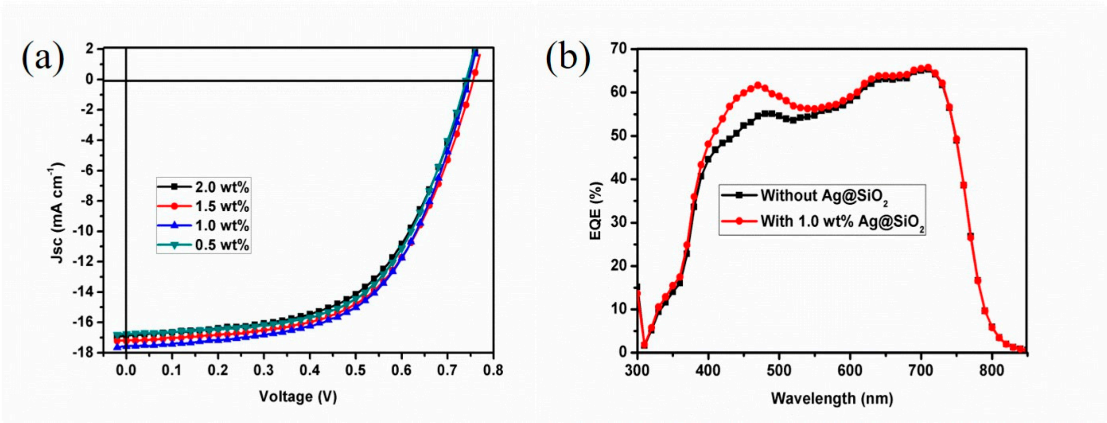

Figure S2. (**a**) I-V curves of PSC devices using different concentrations of Ag–SiO_2 NPs incorporated in the photoactive layer, (**b**) Comparison of EQE spectra of a PSC device applying the optimized incorporation of NPs and a NP-free PSC device.
